# Comment to the Article “Association of Sarcopenia with Pain and Disability in Hand Osteoarthritis: A Retrospective, Cross-Sectional, Single-Center Study”

**DOI:** 10.5152/ArchRheumatol.2026.26335

**Published:** 2026-06-03

**Authors:** Selda Çiftci İnceoğlu

**Affiliations:** Department of Physical Medicine and Rehabilitation, Şişli Hamidiye Etfal Training and Research Hospital, Health Sciences University, İstanbul, Türkiye

Dear Editor,

The study titled “Association of Sarcopenia with Pain and Disability in Hand Osteoarthritis: A Retrospective, Cross-Sectional, Single-Center Study” by Lee et al^^1^^ is quite intriguing and evidently well researched. However, there are a few points where further contributions could be made.

This study evaluates the relationship between radiologically diagnosed hand osteoarthritis and sarcopenia. However, it is not specified whether the radiographic assessments were performed by a single evaluator or by multiple observers. If multiple evaluators were involved, the effect of inter-observer variability should be addressed in the limitations section, and the inclusion of established classification criteria for hand osteoarthritis^^2^^ could have more accurately defined the study population.

Sarcopenia is a skeletal muscle disease that has been extensively investigated in recent years for its associations with various clinical conditions, with numerous publications still emerging regarding its diagnostic process. Although dual-energy absorptiometry and bioelectrical impedance analysis devices are commonly used in clinical practice for diagnosing sarcopenia,^^3^^ evaluating anterior thigh muscle thickness via ultrasonography appears more suitable for the direct assessment of skeletal muscle mass.^^4^^ Additionally, diagnosing sarcopenia requires not only assessing muscle mass but also evaluating physical performance and muscle strength.^^3^^^,^^^4^^ The study appropriately notes the absence of grip strength assessment as a limitation. However, in patients with hand osteoarthritis, the chair stand test could have been evaluated instead of grip strength. Considering all these factors, it seems more appropriate to use the term “low muscle mass” instead of sarcopenia in this study. Furthermore, using national or more current threshold values ​​for the assessment of skeletal muscle mass may be more suitable.

When using the Disabilities of the Arm, Shoulder, and Hand Questionnaire scores for disability assessment, it should be considered that these scores may be influenced by other upper extremity problems.^^5^^ Including whether the patients had additional upper extremity issues in the demographic data would have been more informative. Furthermore, including objective hand function/performance tests in addition to patient-reported questionnaires in this study could have made the functional assessment more comprehensive.

In light of this substantial, labor-intensive study encompassing a large patient cohort, the foregoing observations are proffered constructively, with the aim of guiding and refining the methodological framework of subsequent research endeavors in this domain.

## Data Availability Statement:

The data that support the findings of this study are available on request from the corresponding author.

## Artificial Intelligence Usage Statement:

The author declared that no Artificial Intelligence tool was used in the preparation of the manuscript.

## Peer-review:

Externally peer-reviewed.

## Author Contributions:

Concept – S.Ç.İ.; Design – S.Ç.İ.; Supervision – S.Ç.İ.; Resources – S.Ç.İ.; Materials – S.Ç.İ.; Data Collection and/or Processing – S.Ç.İ.; Analysis and/or Interpretation – S.Ç.İ.; Literature Search – S.Ç.İ.; Writing – S.Ç.İ.; Critical Review – S.Ç.İ.

## Declaration of Interests:

The author has no conflicts of interest to declare.

## Funding:

The author declares that this study received no financial support.
